# Topical compound acid peeling remodels scalp microbiota and lipid homeostasis in seborrheic dermatitis: a multi-omics study

**DOI:** 10.3389/fmicb.2026.1898146

**Published:** 2026-08-26

**Authors:** Hongyan Zhang, Yetan Shi, Lifang Hu

**Affiliations:** 1 Department of Dermatology, Hangzhou Third People’s Hospital, Hangzhou, Zhejiang, China; 2 The Fourth Clinical Medical College, Zhejiang Chinese Medical University, Hangzhou, Zhejiang, China

**Keywords:** compound acid peeling, efficacy, lipidome, *Malassezia*, microbiome, safety, scalp seborrheic dermatitis, *Staphylococcus*

## Abstract

**Background:**

Current therapeutic options for scalp seborrheic dermatitis (SSD) remain suboptimal, highlighting the demand for more effective and safe treatment strategies.

**Objective:**

To investigate the efficacy and underlying mechanisms of a novel 7.5% five-acid compound formulation for the treatment of SSD.

**Methods:**

A total of 319 patients diagnosed with SSD at the dermatology outpatient clinic were enrolled. All patients received four sessions of scalp compound acid therapy and were included in efficacy and safety analyses. A subgroup of 30 male participants from the 319-patient cohort underwent scalp sampling before and after treatment for combined microbiomic and lipidomic detection. Meanwhile, 10 matched male SSD patients who did not received compound acid therapy were enrolled as the control group for multi-omics analyses.

**Results:**

All 319 SSD patients presented marked relief of scalp erythema, scaling and pruritus after repeated compound acid treatment, with only 6 individuals experiencing temporary mild scalp irritation after the initial treatment. Integrated microbiomic and lipidomic analyses were restricted to a male subcohort (30 treated patients and 10 matched untreated male SSD controls). Within this male subgroup, the relative abundance of the genus *Staphylococcus* and the genus *Malassezia* were significantly lowered post-treatment. And the observed lipid alterations may be linked to changes in antioxidant response, inflammatory signaling and fatty acid metabolic pathways. Lipidomic profiling suggests that compound acid peeling may rebalance scalp lipid profiles, accompanied by elevated levels of barrier-protective antioxidant phosphatidylcholines, reduced pro-inflammatory oxidized fatty acids, and altered core fatty acid biosynthesis pathways.

**Conclusion:**

The topical scalp compound acid therapy effectively alleviates clinical symptoms in SSD patients and represents a promising novel therapeutic approach.

## Introduction

Seborrheic dermatitis (SD) is a common chronic relapsing inflammatory skin disorder, mainly affecting sebum-rich areas like the scalp (scalp seborrheic dermatitis, SSD) with erythema, scaling, and pruritus (Adalsteinsson et al., 2020). Despite being underestimated clinically, its high prevalence and quality-of-life impact underscore the need for effective management (Clark et al., 2015). Current first-line pharmacological treatments, primarily azole antifungal agents and topical corticosteroids, are often limited by variable long-term efficacy and safety concerns that restricted continuous use, thus highlighting a pressing need for alternative therapeutic strategies (Jackson et al., 2024).

The complex pathogenesis of SSD involves a triad of factors: *Malassezia* yeast overgrowth, host immune response dysregulation, and a resulting altered scalp lipid composition. Effective treatment must therefore target these multiple etiological components. Chemical peels (chemoexfoliation), a widely used dermatologic procedure, uniquely target multiple SSD pathogenic features simultaneously. A tailored complex acid formulation leverages synergistic properties of its components: Azelaic acid: well-documented anti-inflammatory/antioxidant effects, effective in inflammatory conditions (e.g., papulopustular rosacea, acne vulgaris) (Searle et al., 2022; Webster, 2000); Salicylic acid: potent keratolytic (removes scale/dandruff) with mild anti-inflammatory action and sebum-lowering effects to alleviate scalp pruritus/inflammation (Ekinci et al., 2011; Lu et al., 2019); Glycolic acid (fruit acid): small molecular mass, strong skin penetration, reduces keratinocyte adhesion/accumulation, corrects follicular epithelial keratinization (Arif, 2015; Zhang et al., 2022); Mandelic acid (lipophilic alpha-hydroxy acid): established antibacterial/anti-inflammatory properties (Dayal et al., 2020); Lactobionic acid: gentle topical exfoliant with hydrating, antioxidant, and skin-barrier-repairing effects (Zaenglein et al., 2016). The present study utilized a 7.5% five-acid compound preparation to treat scalp seborrheic dermatitis (SSD). The primary evaluation of clinical efficacy and safety adopted a single-arm design. For multi-omics comparative analysis, 30 treated participants were selected from the overall clinical cohort, and an additional 10 matched patients who did not receive the compound acid therapy were recruited as an observational control group. Collectively, this work offers a promising clinical strategy for SSD intervention.

## Materials and methods

### Patients

Patients diagnosed with scalp seborrheic dermatitis (SSD) at the Dermatology Outpatient Department, Hangzhou Third People’s Hospital, Hangzhou, Zhejiang, China, were consecutively enrolled in this study. The exclusion criteria were as follows: (1) patients with severe internal organ diseases; (2) use of topical antifungals, antifungal shampoos (e.g., ketoconazole), or selenium sulfide lotions within 4 weeks prior to enrollment; (3) application of topical corticosteroids or calcineurin inhibitors within 4 weeks prior to enrollment; (4) presence of infection or unhealed wounds on the scalp; (5) any systemic treatment including corticosteroids, other immunosuppressants or immunomodulators within the past 3 months; (6) comorbid psoriasis, eczema, lichen planus, or other inflammatory dermatoses; (7) pregnancy or lactation.

### Treatment protocol

This study adopted a commercially available novel 7.5% five-acid compound gel is a commercial medical product from Ximeien Technology Co., Ltd., routinely used in our dermatology clinic for scalp seborrheic dermatitis. Each tube contains 14 g gel. Active acidic ingredients: 2% salicylic acid, 1.5% azelaic acid, plus a total 4% mixture of glycolic, mandelic and lactobionic acid. The individual concentrations of the three blended hydroxy acids are proprietary confidential information of the manufacturer and cannot be disclosed due to intellectual property restrictions. The finished formulation is adjusted to pH 3.8. Piroctone olamine serves as the broad-spectrum bacteriostatic agent, so no additional preservatives are added. Poloxamer and polyacryloyl dimethyl taurate act as gel-forming surfactants; glycerin is included as a humectant. No fragrance is incorporated into the product. Combined with suitable excipients, the final product was prepared as a standardized scalp compound acid preparation. Each patient received an identical dose of the compound acid formulation, which was applied to cover the entire scalp.

Patients in the treatment group received in-hospital administration of the 7.5% five-acid compound scalp formulation once weekly (at a fixed 7-day interval), for a total of 4 consecutive treatment sessions. Prior to each treatment session, standardized clinical photographs of the target lesion sites were obtained. Disease severity scoring was performed by two independent board-certified dermatologists using a modified “16-point scale (Zhang et al., 2023)”. To achieve more precise differentiation of clinical manifestations, we refined the grading standards for pruritus. The scale comprises three subdomains: erythema scored from 0 to 3 points, scaling scored from 0 to 10 points, and pruritus scored from 0 to 9 points, with an overall total score spanning 0 to 22 points. Full detailed scoring descriptors for each grade of erythema, scaling and pruritus are presented in Table 1. When discrepancies in the severity grading of scalp seborrheic dermatitis occurred between the two evaluators, a third independent board-certified dermatologist was consulted to make the final assessment. All adverse events occurring throughout the treatment period were documented concurrently, with severity graded as mild (transient discomfort, no treatment required), moderate (persistent symptoms > 24 h, symptomatic treatment required), or severe (severe irritation, treatment interruption required).

**TABLE 1:** Modified severity scoring scale for scalp seborrheic dermatitis.

Symptom	Scoring range	Score	Clinical description
Erythema	0–3 points	0	No erythema
		1	Erythema area ≤ 2 cm^2^
		2	2 cm^2^ < erythema area < 5 cm^2^
		3	Erythema area ≥ 5 cm^2^
Scaling	0–10 points	0	No scalp flaking
		1–2	Small powdery grayish coarse scales
		3–4	Small to moderate flakes
		5–6	Large and thin flakes loosely attached to the scalp
		7–8	Adherent flakes
		9–10	Thicker white- yellow scales closely attached to the scalp
Pruritus	0–9 points	0	No pruritus.
		1–3	Mild pruritus, tolerable, no impact on daily activities or sleep.
		4–6	Moderate pruritus, requires scratching/massage relief, partial impact on daily life/sleep.
		7–9	Severe pruritus, incessant, significant disruption to daily activities and sleep.
Total score	0–22 points	–	Sum of erythema, scaling and pruritus scores; higher score = more severe SSD

This modified scale was adapted from the clinical evaluation criteria for scalp seborrheic dermatitis (Zhang et al., 2023), with an expanded pruritus scoring system for more precise quantification of clinical symptoms.

The standardized administration protocol of the 7.5% five-acid compound scalp formulation was as follows: all applications were performed by two professionally trained and certified nurses wearing sterile gloves, who spread the formulation evenly over the entire scalp from the forehead to the occiput. Each patient was administered one tube of the compound acid formulation with a fixed total weight of 14 g. The preparation was evenly spread across the entire scalp, with an extra uniform coating applied over regions with prominent lesions. The total dosage was fixed and not adjusted according to hair length. The allowable residence time window of the product on the scalp was defined as 4–6 h. The actual residence time of every single treatment session for each subject was fully documented. After the specified residence time, patients performed self-cleansing of the scalp at home using a mild, non-medicated shampoo free of active pharmaceutical ingredients. Participants were retained in the final clinical assessment, including those who complied with the 4–6 h residence standard for all four sessions, as well as individuals who only failed to meet the standard residence time during the first treatment but complied within the required range for the following three treatments.

### Grouping information

In this single-arm clinical trial, a total of 319 patients with scalp seborrheic dermatitis completed four cycles of standardized five-acid peeling therapy. All clinical efficacy and safety data presented were derived from this full large cohort. For exploratory multi-omics mechanistic analysis, we strictly screened 30 eligible male subjects from the 319-patient cohort who visited our dermatology outpatient department between September and November according to the modified 16-point scale inclusion criteria: aged 18–45 years, erythema lesion area ranging from 2 cm^2^ to 5 cm^2^, large thin flakes loosely attached to the scalp, and mild tolerable pruritus that did not interfere with daily activities or sleep. These screened participants constituted the omics treatment group and received the identical compound acid intervention as the overall cohort. Meanwhile, we recruited another 10 male SSD patients who attended the outpatient department within the same September–November timeframe and were well-matched with the 30 omics-treated males in age and disease severity as an independent observational control group. Subjects in the control group received no five-acid peeling intervention and only used mild non-medicated shampoo for daily scalp cleansing. Microbiome sequencing was carried out in both the 30 treated males and 10 matched control males, while lipidomic profiling was only conducted on samples collected from the 30 screened treated subgroup due to exploratory research limitations. Of note, only male patients were selected for multi-omics detection to eliminate gender-related confounding factors. Females were excluded because frequent diverse hair-care products and routines would introduce uncontrollable interference to scalp microbiome and lipid profiles, while male scalp samples present higher baseline homogeneity to ensure stable and comparable omics data.

### Scalp microbiome sample collection

Scalp sebum samples were collected at two time points, treatment group: baseline (before treatment) and after the 3rd treatment session. Control group: baseline and day 21. The sampling time points for scalp lipid collection were identical between the two groups. Microbiological analysis was performed in both the treatment and control groups, while lipidomic analysis was conducted only in the treatment group. All subjects were instructed to refrain from hair washing for 48 h before sample collection. For scalp sampling, D-Squame tapes were applied for 3 min to the designated areas. Specifically, sampling was performed at three distinct anatomical locations on the vertex of the scalp. At each location, two tape strips were taken sequentially from the exact same site, resulting in a total of six strips per participant. Prior to extraction, all six strips from each participant were pooled, finely minced, and thoroughly mixed to create a single representative sample. This pooled mixture was then divided into two equal portions for subsequent microbiome and lipidome analyses, respectively. All scalp samples were collected in the dermatology outpatient clinic of the hospital in Hangzhou, Zhejiang Province, from September to November. Central air conditioning operated throughout the sampling period to maintain a constant indoor environment: temperature 24–26 °C and relative humidity 40%–60%. The indoor temperature and humidity were monitored daily during sample collection to minimize environmental interference with scalp microbiome and lipid profiles.

### DNA extraction and sequencing library preparation

Genomic DNA was extracted from samples using DNA Mini Kit (Magen, China). DNA quality was verified via 1% agarose gel electrophoresis, and quantified with Qubit 4.0 Fluorometer and NanoDrop 2000 Spectrophotometer. For PCR: bacterial 16S rRNA V3-V4 region was amplified with primers 338F (5′-ACTCCTACGGGAGGCAGCAG-3′) and 806R (5′-GGACTACHVGGGTWTCTAAT-3′); fungal ITS region with primers ITS1F (5′-CTTGGTCATTTAGAGGAAGTAA-3′) and ITS2R (5′-GCTGCGTTCTTCATCGATGC-3′). Purified amplicons were used to construct Illumina libraries (per standard procedures, TransStart FastPfu PCR SuperMix), then sequenced on Illumina NovaSeq 6000 (paired-end PE250).

### Sequencing and bioinformatics analysis of 16S rRNA and ITS sequences

Purified PCR amplicons were size-selected (AxyPrep DNA Gel Extraction Kit) and quantified (Qubit 4.0). Libraries were constructed with 2 × TransStart FastPfu PCR SuperMix (Illumina standard SOP) and sequenced on NovaSeq 6000 PE250 platform (Illumine, San Diego, United States). Raw data were quality-filtered and adapter-removed via fastp v0.20.0 (3′-truncation: 50 bp sliding window, Phred <20; read merging: ≥10 bp overlap). Sequences were clustered into operational taxonomic units (OTUs) at a 97% similarity threshold using Vsearch software (version 2.22.1) to generate OTUs and the corresponding feature table. Taxonomic classification of representative OTU sequences was performed with the RDP Classifier (version 2.13) at a confidence cutoff of 0.7. The SILVA 138.1 database was used for taxonomic annotation of bacterial sequences, while the UNITE 9.0 database was applied for fungal sequences.

For both 16S rRNA (bacteria) and ITS (fungi) amplicon sequencing, extraction blank controls and PCR negative controls were processed synchronously with scalp samples during DNA isolation, PCR amplification and library construction, and subjected to identical sequencing procedures to identify and eliminate reagent and environmental microbial contaminants. All contaminant taxa detected in blank controls were filtered out prior to bioinformatic analysis. Each sample was sequenced to a target depth of ≥50,000 clean reads for 16S rRNA and ≥40,000 clean reads for ITS. Low-quality reads, adapters and chimeric sequences were discarded to obtain valid clean reads. To normalize uneven sequencing depth across samples, all datasets were rarefied to the minimum read count within the cohort, and rarefaction curves were plotted to confirm sequencing saturation. Relative abundance of each taxon was calculated based on rarefied read counts. For intergroup differential abundance testing, centered log-ratio (CLR) transformation was applied to correct compositional bias of amplicon data.

### Lipid extraction

Lipids were extracted from D-Squame^®^ tapes using a modified Methyl tert-butyl ether (MTBE) method. The sample was homogenized following the addition of 300 μL of pre-chilled methanol/water (2:1, v/v). Subsequent lipid phase separation was achieved by introducing 600 μL of MTBE, followed by 10 min of ultrasonication in an ice bath. The mixture was centrifuged (14,000 × *g*; 4 *^o^*C, 10 min), and the upper lipid-rich organic layer was collected. The solvent was removed under vacuum, and the resulting dried lipid samples were stored at −80 *^o^*C until analysis.

### Lipidomic analysis

Lipidomic analysis used Nexera UHPLC LC-30A + high-resolution Orbitrap MS. Raw data were preprocessed via Progenesis QI V2.0 (peak alignment, normalization), with compound identification validated against LIPID MAPS. After quality filtering/imputation, data underwent z-score normalization. Orthogonal Partial Least Squares Discriminant Analysis (OPLS-DA; predI = 1, orthoI = 1, scaleC = “standard”, og10L = TRUE, permI = 200) for pattern recognition (VIP scores for variable importance). KEGG pathway enrichment used hypergeometric test. Fold changes (FC) = inter-group mean abundance ratios. Significant features (for further analysis): VIP ≥ 1.0, |log_2_(FC)| ≥ 0.263 (FC ≥ 1.2/≤0.833), *P* < 0.05.

### Statistical methods

Statistical analyses were performed in R4.1.3 with packages vegan (v2.6-4), phyloseq (v1.38.0), ggpubr (v0.5.0) and other supporting packages. Shapiro-Wilk test was applied to assess the normality of continuous variables.

For within-subject comparisons (pre- vs. post-treatment in the 30 treated males): paired samples *t*-test was used for normally distributed data, and Wilcoxon signed-rank test for non-normal data. For intergroup comparisons between treated males and untreated male controls, unpaired Wilcoxon rank-sum tests were adopted.

Linear discriminant analysis effect size (LEfSe) was used to screen differential taxa across groups (Kruskal–Wallis *P* < 0.05, log LDA score ≥ 2.0). Spearman’s rank correlation was calculated to analyze associations between microbial taxa. Differential lipid metabolites were identified via Student’s *t*-test, fold change (FC) and variable importance in projection (VIP) values from OPLS-DA.

The Benjamini-Hochberg (BH) method was applied to correct *P*-values for all multiple taxonomic and lipidomic comparisons to control false discovery rate. All statistical tests were two-sided with α = 0.05; adjusted *P* < 0.05 was defined as statistically significant.

For clinical symptom score changes in the full 319-patient cohort, Cohen’s d (standardized mean difference) was calculated as the effect size, and 95% confidence intervals (95% CIs) were reported for primary and secondary clinical endpoints to quantify therapeutic efficacy.

## Results

**FIGURE 1:**
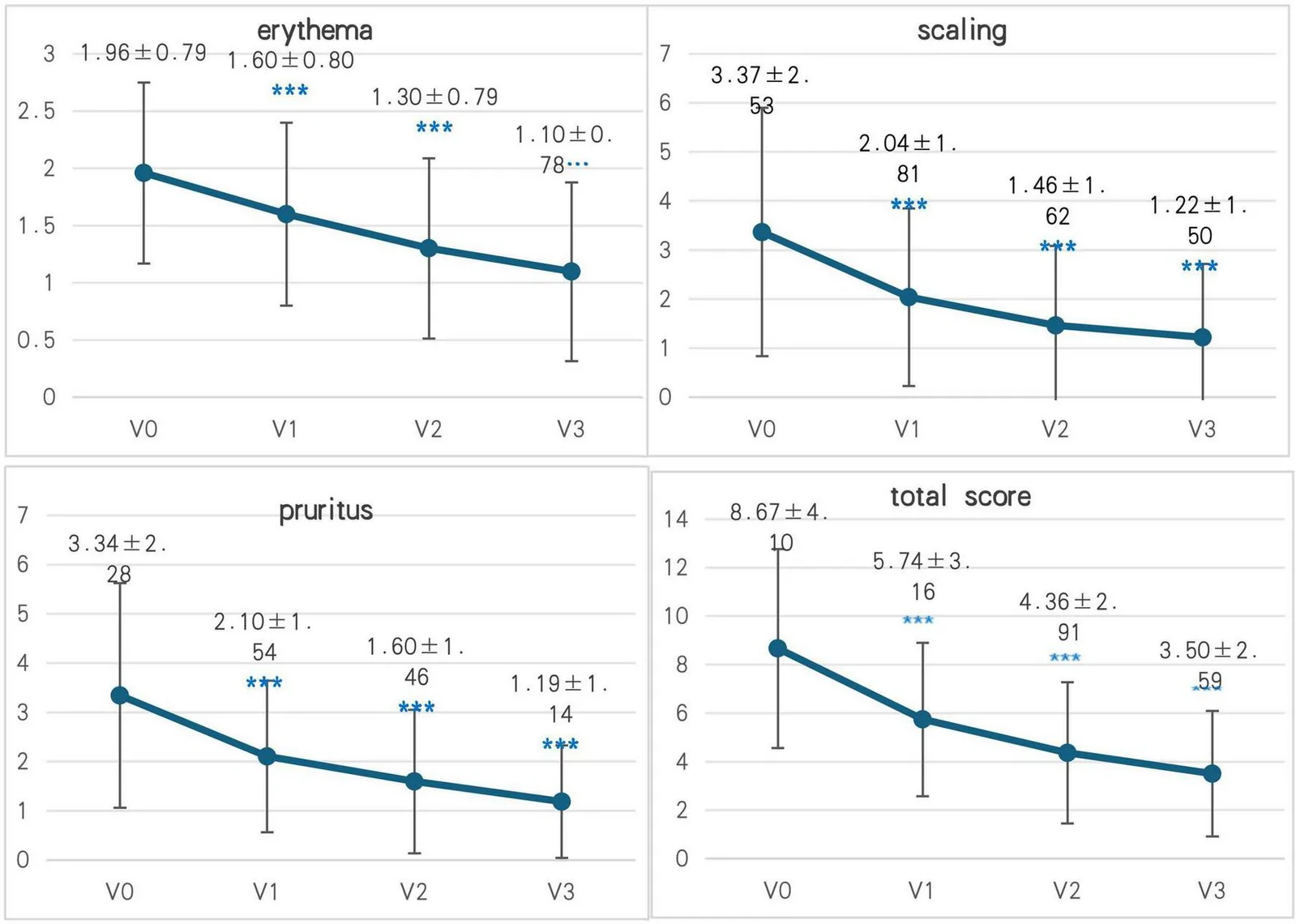
After each treatment with scalp compound acid, significant differences were observed in the scores for scalp erythema, scales, itching, and the total score (*n* = 319, ***P < 0.001).

A total of 319 patients (155 men [48.6%] and 164 women [51.4%]) completed four consecutive compound acid peeling sessions, and all clinical efficacy data presented in Figure 1 were generated based on this full cohort. For multi-omics mechanistic research, 30 male patients were screened from these 319 individuals as the treatment subgroup, and an extra 10 matched male SSD patients without acid intervention served as the control group for microbiome analysis, with lipidomic testing restricted to the 30 treated males (Table 2).

**TABLE 2:** Baseline demographic and clinical characteristics of the study populations.

Characteristics	Total clinical cohort (*N* = 319)	Sub-study treatment group (*n* = 30)	Sub-study control group (*n* = 10)
Age (years, mean ± SD)	29.8 ± 9.4	**31.5 ± 8.2**	29.1 ± 6.8
Gender (male / female)	155 (48.6%) / 164 (51.4%)	30 (100%) / 0 (0%)	10 (100%) / 0 (0%)
**Fitzpatrick skin type**			
- Type III	121 (37.9%)	11 (36.7%)	4 (40.0%)
- Type IV	198 (62.1%)	19 (63.3%)	6 (60.0%)
Disease duration (months)	14.2 ± 6.8	13.5 ± 5.9	14.0 ± 6.2
Baseline severity score	8.67 ± 4.10	8.54 ± 3.85	8.60 ± 3.90

Bold values indicate Fitzpatrick skin type: a classification system evaluating skin susceptibility to ultraviolet radiation.

### Efficacy of topical scalp complex acid

A total of 319 patients (155 men [48.6%] and 164 women [51.4%]) with SSD completed 4 sessions of the compound acid treatment. The mean age was 29.8 ± 9.1 years (range 11–49 years) for male patients and 29.9 ± 9.7 years (range 12–61 years) for female patients. At the initial diagnosis, the mean scores for SSD were recorded as follows: erythema 1.96 ± 0.79 points, scaling 3.37 ± 2.53 points, and pruritus 3.34 ± 2.28 points, with a total score of 8.67 ± 4.10 points. After the first treatment session, significant reductions were observed in erythema, scaling, itching, and total severity scores, with incremental improvements noted as treatment sessions progressed. Assessments conducted prior to the fourth session demonstrated further declines in erythema 1.10 ± 0.78 points, scaling 1.22 ± 1.50 points, and pruritus 1.19 ± 1.14 points, with a total score of 3.50 ± 2.59 points (Figure 1). Marked improvement in skin lesions was also observed clinically (Supplementary Figures 1–3).

### Safety and tolerability of topical scalp complex acid

Adverse reactions were mild, occurring in 6 cases out of a total of 319 patients who underwent acid treatment. The reaction was mild scalp irritation, which was observed in patients with significant scalp itching and scratching. However, the irritation was transient and resolved after washing. It occurred during the first application and disappeared during subsequent treatments.

### Scalp seborrheic dermatitis bacterial diversity and composition

**FIGURE 2:**
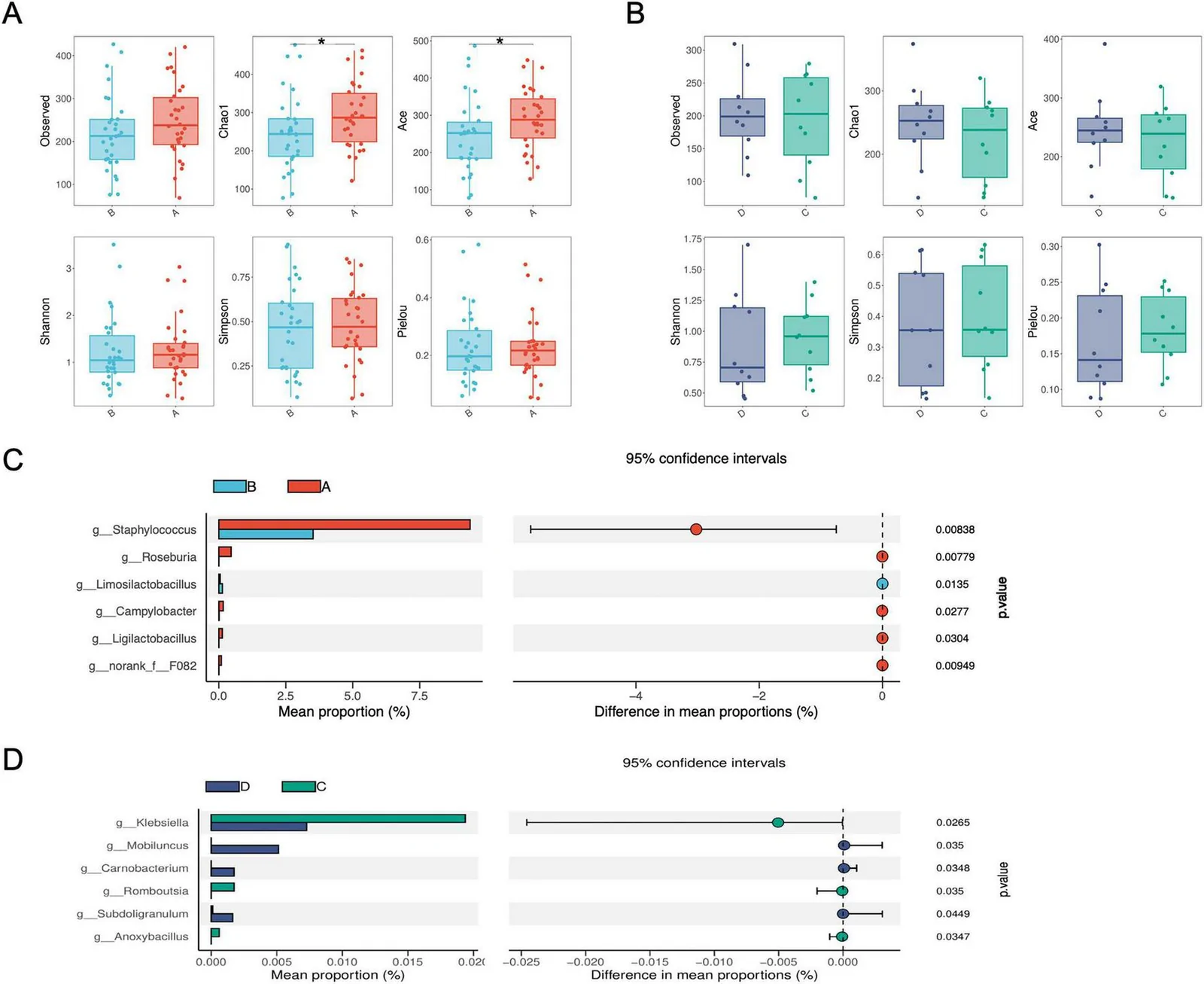
Scalp bacterial diversity decreased after treatment with scalp compound acid. **(A)** Bacterial α-diversity box-plot of treatment group. **(B)** Bacterial α-diversity box-plot of control group. **(C)** Top 6 bacterial species differences of treatment group. **(D)** Top 6 bacterial species differences of control group. Group A: Before treatment with scalp compound acid in treatment group, Group B: After treatment with scalp compound acid in treatment group, Group C: Before no treatment with scalp compound acid in control group, Group D: After no treatment with scalp compound acid in control group (treatment group A,B: *n* = 30, control group C,D: *n* = 10, *P < 0.05).

To evaluate the impact of the acid regimen on SSD, we assessed α-diversity and bacterial taxonomic composition (Figure 2). Post-treatment, the treatment group exhibited a significant reduction in overall bacterial α-diversity compared to the pre-treatment baseline. Specifically, two richness metrics, the Chao1 and ACE indices, were statistically significantly decreased (*P* < 0.05 for both, Figure 2A). In sharp contrast, the control group showed no significant differences in any α-diversity metric over the same period (Figure 2B). However, the abundance of *Staphylococcus* and *Roseburia* decreased significantly in the treatment group after therapy (*P* < 0.05) (Figure 2C). Neither *Staphylococcus* nor *Roseburia* showed any significant change in the control group (Figure 2D).

### Scalp seborrheic dermatitis fungal diversity and composition

**FIGURE 3:**
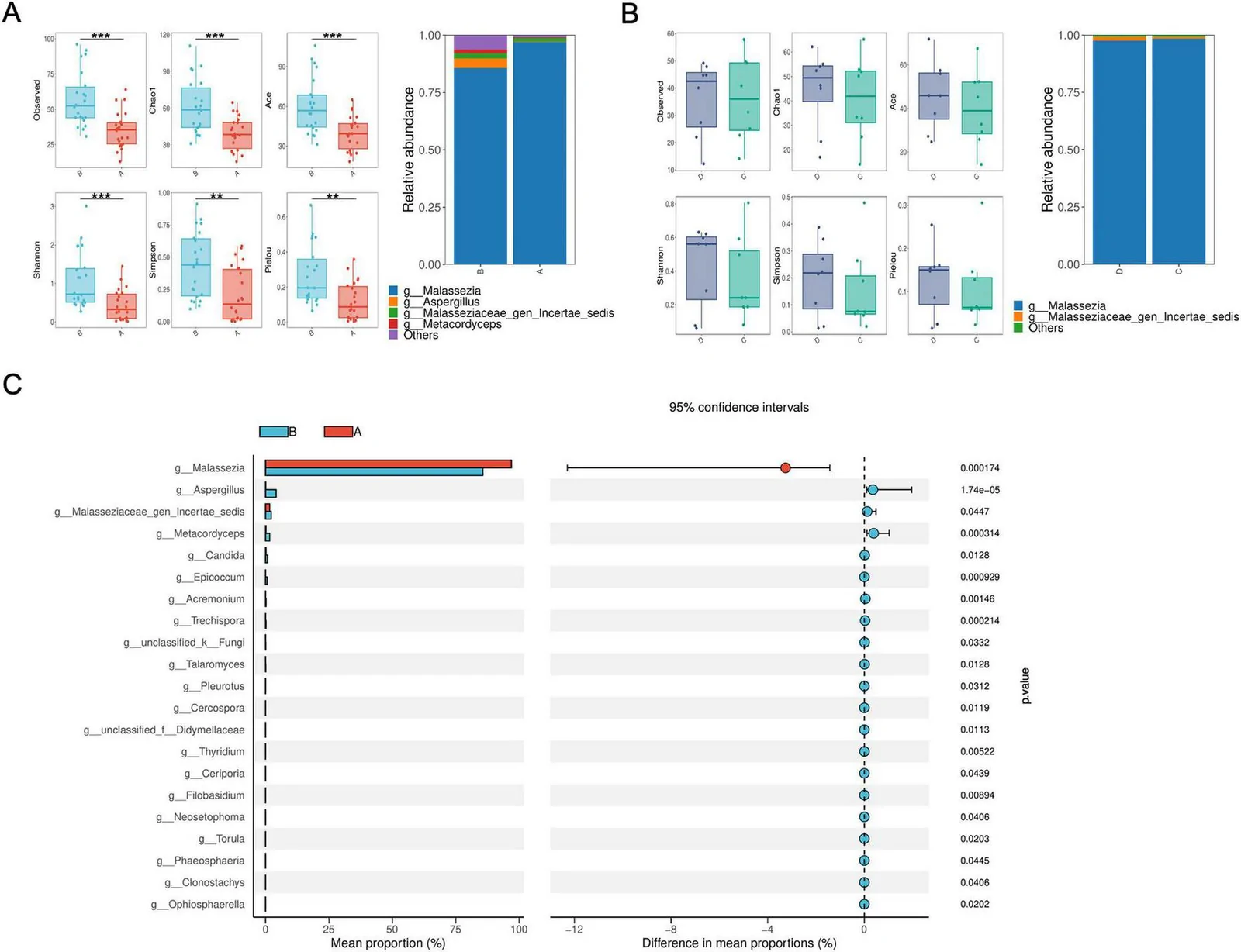
Fungal diversity increased after treatment with scalp compound acid. **(A)** Fungal α-diversity box-plot (left) and community bar (right) of treatment group. **(B)** Fungal α-diversity box-plot (left) and community bar (right) of control group. **(C)** Fungal species differences of treatment group. Group A: Before treatment with scalp compound acid in treatment group, Group B: After treatment with scalp compound acid in treatment group, Group C: Before no treatment with scalp compound acid in control group, Group D: After no treatment with scalp compound acid in control group (treatment group A,B: *n* = 22, control group C,D: *n* = 8, *P < 0.05, **P < 0.01, ***P < 0.001).

ITS sequencing revealed that acid peeling induced a dramatic and comprehensive increase in fungal α-diversity in the treatment group. All six tested α-diversity metrics (Chao1, ACE, Shannon, etc.) were statistically significantly elevated post-treatment (Figure 3A). Conversely, the control group showed no significant changes (Figure 3B). Analysis of community structure showed that the dominant genus, *Malassezia*, was significantly decreased in relative abundance in the treatment group post-therapy (*P* < 0.05, Figures 3A,B). This suppression of *Malassezia* was accompanied by a significant expansion of non-*Malassezia* genera, including *Aspergillus* and *Metacordyceps* (Figure 3C). By contrast, the control group exhibited only differential changes in non-dominant taxa. This suggests that acid peeling may significantly increase overall fungal diversity by suppressing the dominant commensal (the genus *Malassezia*) and promoting the expansion of other taxa.

### Differences in scalp surface lipidomic profile

**FIGURE 4:**
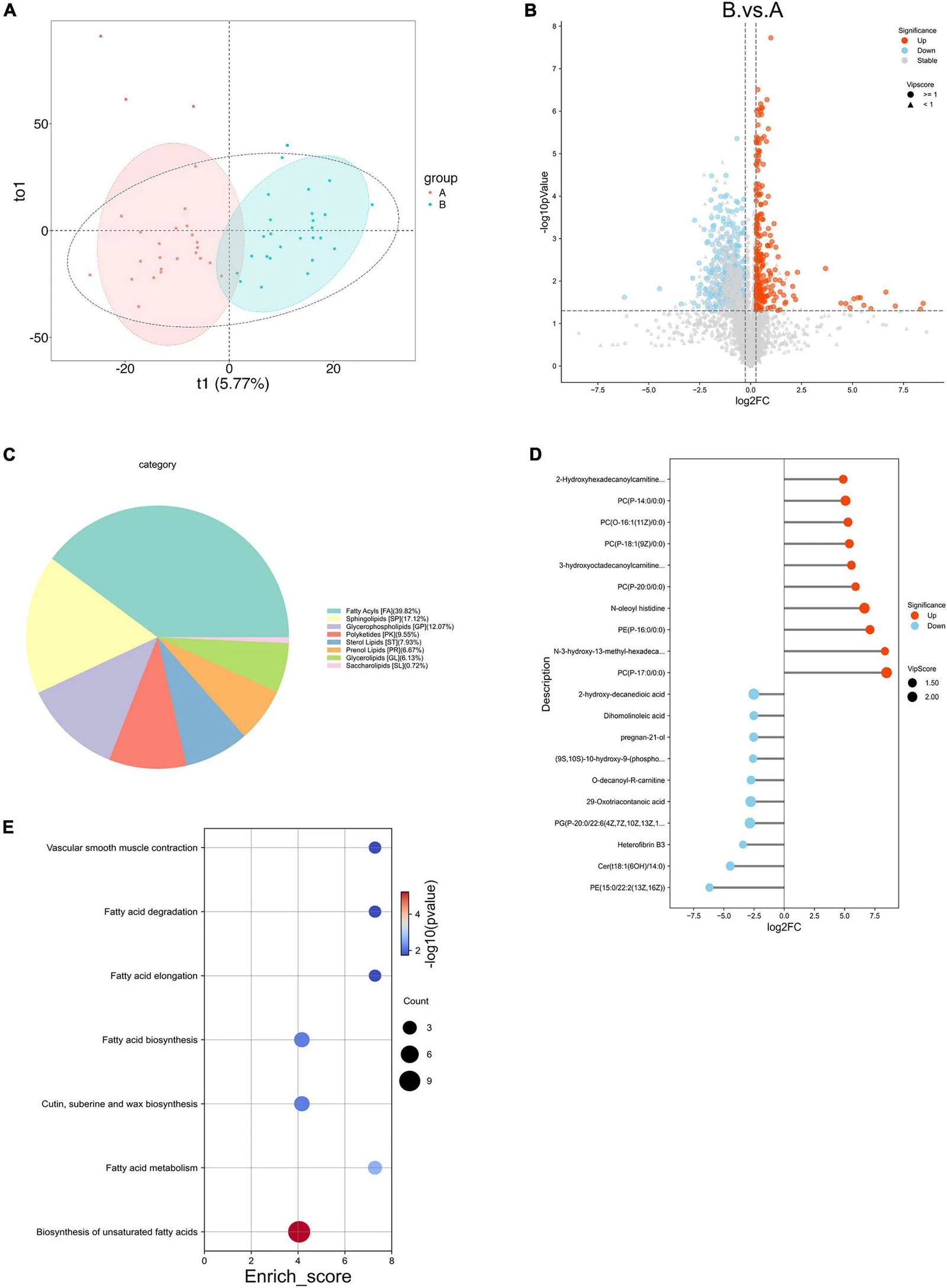
Alterations in lipid of scalp surface treatment with scalp compound acid. **(A)** OPLS-DA score plot between Group A and Group B. **(B)** Volcano plot between Group A and Group B. **(C)** Lipid analysis showed that the differences were primarily from fatty acids. **(D)** Distribution map of VIP values of lipid differential metabolites with scalp compound acid. **(E)** KEGG pathway enrichment of lipid differential metabolites with scalp compound acid. Group A: Before treatment with scalp compound acid in treatment group, Group B: After treatment with scalp compound acid in treatment group (treatment group A,B: *n* = 30).

Lipidomic profiling was performed on paired pre- and post-treatment samples from the treatment group to characterize lipid alterations following compound acid peeling. The OPLS-DA score plot exhibited clear separation between pre-treatment and post-treatment lipid profiles (Figure 4A). Volcano analysis identified numerous differentially expressed lipid species after treatment (Figure 4B). Classification analysis of differential lipids indicated that fatty acid-related lipids constituted the primary differential components driving the overall lipid profile changes (Figure 4C). Further analysis of significantly altered lipids revealed distinct expression changes after treatment (Figure 4D). A set of phosphatidylcholine (PC) species, including P-17:0/0:0, P-20:0/0:0, P-18:1(9Z)/0:0, and O-16:1(11Z)/0:0, were significantly upregulated after intervention (*P* < 0.05). Acylcarnitines (including 2-hydroxyhexadecanoylcarnitine, 3-hydroxyoctadecanoylcarnitine, and N-3-hydroxy-13-methyl-hexadecanoylcarnitine) and N-oleoyl histidine were also significantly upregulated after treatment. Acylcarnitines are involved in mitochondrial transport and oxidation of fatty acids. Their accumulation may reflect the acceleration of the oxidative metabolism of fatty acids or changes in their transport (Chintapalli et al., 2016; Li et al., 2019). N-oleoyl histidine belongs to N-acyl compounds and is generally classified as a long-chain N-acyl amino acid. N-oleoyl histidine is an endogenous substance and may have neuroregulatory or anti-inflammatory effects (Burstein et al., 2012). In contrast, dihomolinoleic acid was downregulated after treatment. Moreover, ceramide (Cer) and phosphatidylethanolamine (PE) species showed decreased expression following compound acid intervention. KEGG pathway enrichment analysis was conducted based on all significantly changed lipid metabolites (Figure 4E). The downregulated differential lipids were most significantly enriched in the pathway of “Biosynthesis of unsaturated fatty acids.” Multiple core fatty acid metabolic pathways, including fatty acid synthesis, elongation, and degradation pathways, exhibited overall downregulated trends after treatment.

## Discussion

Scalp seborrheic dermatitis (SSD) is a common chronic inflammatory scalp disorder, causing persistent itching, scaling, erythema, and greasiness that impair mental health and quality of life (Searle et al., 2022). Its multifactorial etiology involves dysregulated sebaceous gland function, microbial dysbiosis (notably overgrowth of *Malassezia* species), and inflammatory cascades triggered by irritating free fatty acids from hydrolyzed sebum (Jackson et al., 2024). Current standard treatments (topical azoles, corticosteroids, calcineurin inhibitors) often lack sustained efficacy, with high relapse rates, antifungal resistance risks, and local tolerability issues (e.g., cutaneous atrophy from prolonged corticosteroid use) undermining long-term adherence (Naldi and Rebora, 2009). Our study assesses a topical compound acid formulation as a novel, multi-mechanistic therapy. Its clinical efficacy, enhanced with successive treatments, addresses multiple pathogenic pathways to overcome monotherapy limitations, supporting potential for SSD stability and good tolerability.

The acid peel regimen restructured the scalp microenvironment and relieved SSD symptoms including erythema, scaling, greasiness and pruritus. Microbiome analysis showed a selective reversal of dysbiosis: the acid treatment led to a significant reduction in bacterial α-diversity, and the abundance of the genus *Staphylococcus* was markedly decreased. When compared to existing literature showing increased α-diversity and the genus *Staphylococcus* abundance in patients with seborrheic dermatitis (SD; Maître et al., 2025; Tao et al., 2021), these findings could suggest partial improvement of scalp microbial dysbiosis. Fungal α-diversity was significantly increased, accompanied by a marked relative abundance reduction in the genus *Malassezia*, a primary inflammatory trigger (Yu et al., 2025). Unfortunately, we only performed untargeted microbiome analysis and failed to characterize alterations at the species level. Wang et al. (2022) found that shampoo containing 6% glycyrrhetinic acid complex significantly alleviated scaling lesions and improved the quality of life in patients with scalp seborrheic dermatitis. They further quantified four core scalp microorganisms, namely *Cutibacterium acnes*, *Staphylococcus epidermidis*, *Malassezia restricta* and *Malassezia globosa*, via qPCR. The results demonstrated that the intervention markedly upregulated the abundance of *C. acnes*, reduced the level of *S. epidermidis*, and simultaneously decreased the loads of the two pathogenic *Malassezia* species (Wang et al., 2022). Similar to their study, which demonstrated highly significant reductions in erythema, scaling, and pruritus within a 3-week evaluation window, our 7.5% five-acid compound formulation achieved progressive clinical symptom relief over a matching 21-day period. In terms of safety, while Wang et al. (2022) noted zero adverse events with their therapeutic shampoo, our formulation encountered a very low incidence of mild, transient irritation (6 out of 319 cases), a predictable outcome given the intense chemoexfoliative nature of concentrated alpha/beta-hydroxy acids compared to cleansing vehicles. From a microbiological perspective, although both interventions aim to restore balanced scalp microbiota, our multi-acid peeling treatment delivers rapid antimicrobial activity to lower the relative abundances of the dysbiotic genera *Staphylococcus* and *Malassezia*, which may facilitate swift reconstruction of the scalp microbial microenvironment.

Crucially, Lipidomic analysis suggested alterations in scalp lipid composition, which may indicate potential protective mechanisms. The KEGG pathway enrichment revealed a broad and significant downregulation of fatty acid metabolism. This trend may correlate with improved scalp sebum composition and lipid turnover, which could contribute to alleviating seborrheic dermatitis lesions (Mosca et al., 2025). Strategies for regulating skin lipid metabolism are expected to provide new therapeutic directions for *Malassezia*-related skin diseases (such as SD) (Cheng et al., 2025). This was paired with two key functional shifts in our study: Dihomolinoleic acid is a PUFA (polyunsaturated fatty acid) and a precursor of oxidized fatty acids (OxFAs). A downregulation of pro-inflammatory OxFAs (which may correlate with the relief of erythema and pruritus) and a concurrent upregulation of barrier-protective phosphatidylcholines (PC), which may correlate with improved skin barrier integrity and attenuated oxidative stress. It has been reported that lowered synthesis of pro-inflammatory oxidized fatty acids (OxFAs/eicosanoids) is associated with attenuated cutaneous inflammation and pruritus in inflammatory dermatoses including atopic dermatitis (Sawada et al., 2021). Lipids, especially phospholipids, are essential for skin barrier homeostasis. As a major membrane constituent, phosphatidylcholine (PC) preserves the structural integrity and physiological function of stratum corneum cell membranes (Bouwstra et al., 2023). Decreased levels of ceramides (Cer) and phosphatidylethanolamines (PE) may be associated with altered apoptosis or lipid turnover. Notably, highly unsaturated lipids such as PE tend to accumulate under pathological scalp conditions. Our multi-omics data suggest that compound acid peeling may relieve scalp inflammation and improve barrier function, possibly through reshaping scalp microbial profiles and modulating lipid metabolism.

Several limitations of this study need to be addressed. Firstly, the sample size for multi-omics analysis was limited, and all enrolled participants were male without female subjects. Thus, the conclusions drawn from microbiome and lipidomic profiles cannot be generalized to all patients with scalp seborrheic dermatitis. Secondly, we only performed correlative profiling of microbiome and lipidome, and lipidomic detection was merely conducted in the treatment group. Without supplementary cellular and animal functional experiments, the exact molecular mechanisms by which compound acid regulates the scalp microenvironment cannot be fully clarified. Thirdly, this work was a single-arm clinical observation lacking a parallel independent control group. Large-cohort studies with extended follow-up periods are required to verify long-term recurrence risk and sustained safety profiles.

Future research should carry out large-sample randomized, double-blind, placebo-controlled trials to further validate the efficacy of this compound acid preparation. Extended follow-up periods are also needed to evaluate long-term recurrence risks of scalp seborrheic dermatitis. In addition, purified single acid substances without excipients will be adopted in subsequent *in vitro* and *in vivo* experiments to eliminate the confounding effects of auxiliary ingredients, which can help systematically clarify the precise molecular regulatory mechanisms underlying scalp microecology and lipid homeostasis.

## Conclusion

This single-arm clinical evaluation verifies the favorable efficacy and safety of topical compound acid peeling for scalp seborrheic dermatitis, which substantially improves typical clinical symptoms with only minor temporary adverse reactions. Distinct from the single-arm clinical assessment, a matched-control multi-omics analysis was performed to dissect mechanistic changes. The omics data suggest that the intervention may ameliorate scalp microbial dysbiosis by reducing the relative abundance of the genus *Staphylococcus* and the genus *Malassezia*, and the observed lipid alterations may be linked to changes in antioxidant response, inflammatory signaling and fatty acid metabolic pathways. Overall, compound acid peeling provides a promising candidate intervention for SSD clinical treatment.

## Data Availability

The raw sequence data reported in this paper have been deposited in the Genome Sequence Archive (Genomics, Proteomics & Bioinformatics 2025) in National Genomics Data Center (Nucleic Acids Res 2025), China National Center for Bioinformation / Beijing Institute of Genomics, Chinese Academy of Sciences (GSA: CRA047627 and GSA: CRA048477) that are publicly accessible at https://ngdc.cncb.ac.cn/gsa. Raw amplicon sequencing data were deposited in the Genome Sequence Archive (GSA, CNCB-NGDC) under accession PRJCA070221.
